# Supplementary material for: Data asset valuation model based on generative artificial intelligence
Source: PLoS One. 2025 Aug 18;20(8):e0328926. doi: 10.1371/journal.pone.0328926 (PMC12360564; doi:10.1371/journal.pone.0328926)
Supplement: Attachment — Submitted filename: MH25041901-Response Letter(1).docx [file pone.0328926.s001.docx]

**Response to Reviewers**

**We responded to each issue raised by the reviewers in a bold and underlined manner.**

Reviewers' comments:

1. Is the manuscript technically sound, and does the data support the conclusions?

The manuscript must describe a technically sound piece of scientific research with data that supports the conclusions. Experiments must have been conducted rigorously, with appropriate controls, replication, and sample sizes. The conclusions must be drawn appropriately based on the data presented.

Reviewer #1: Partly

Reviewer #2: Yes

**Response: For Reviewer 1’s response of “Partly”, we summarized what has been conducted. The data asset valuation model is constructed using a Generative AI algorithm based on real data. To construct a robust and practical model, widely adopted machine learning models are used to pick influential predictive variables. The constructed model is tested using validation and test data sets. Also, it is shown that fusion models are more capable than single models.**

2. Has the statistical analysis been performed appropriately and rigorously?

Reviewer #1: Yes

Reviewer #2: Yes

**Response: No responses are required**

3. Have the authors made all data underlying the findings in their manuscript fully available?

The [PLOS Data policy](http://www.plosone.org/static/policies.action#sharing) requires authors to make all data underlying the findings described in their manuscript fully available without restriction, with rare exceptions (please refer to the Data Availability Statement in the manuscript PDF file). The data should be provided as part of the manuscript or its supporting information, or deposited to a public repository. For example, in addition to summary statistics, the data points behind means, medians, and variance measures should be available. If there are restrictions on publicly sharing data—e.g. participant privacy or use of data from a third party—those must be specified.

Reviewer #1: Yes

Reviewer #2: No

**Response: For Reviewer 2’s response of “No”, we provide the link for the data set:** [**https://www.hkex.com.hk/Mutual-Market/Stock-Connect/Eligible-Stocks/A-share-Lookup-Tools?sc_lang=en**](https://www.hkex.com.hk/Mutual-Market/Stock-Connect/Eligible-Stocks/A-share-Lookup-Tools?sc_lang=en)**. However, we do not share the collected and cleansed data set in the article, instead the data set was shared in China macroeconomic database.**

4. Is the manuscript presented in an intelligible fashion and written in standard English?
PLOS ONE does not copyedit accepted manuscripts, so the language in submitted articles must be clear, correct, and unambiguous. Any typographical or grammatical errors should be corrected at revision, so please note any specific errors here.

Reviewer #1: Yes

Reviewer #2: Yes

**Response: No responses are required.**

5. Review Comments to the Author

Reviewer #1: This article builds a generative artificial intelligence-based data asset valuation model based on existing literature, which integrates data feature extraction, a generative value generation algorithm, and market adaptive valuation to dynamically assess the business value of data assets. The conclusions of the article show that the model can address the shortcomings of traditional valuation methods in data asset valuation. Overall, the article is well written, the analysis is solid and the conclusions are challenging and bring a lot of new information to the field.
However, there are certain issues, such as the sample and analysis methods of the study, that deserve in-depth discussion. There are four issues that require further research.

1. What is the basis for the composition of the three dimensions of the model? What are the criteria for dimension selection? Please elaborate. Please elaborate.

**Response: All three dimensions are critically significant in the construction data asset valuation model. Each has a crucial role in refining the contracted data asset valuation model. We do not use any criteria in dimension selection. However, the related literature gives us insight into the dimensions of the model.**

2. Do metrics such as user data, market data, and operational data provide a comprehensive picture of the data assets reported by the companies? A data asset is an asset that is formed from data elements by adding value, not just a collection of data. Please elaborate.

**Response: Yes, user data, market data, and operational data provide a picture for data assets, however, their role is complimentary. Their collection process and accessibility to them are also a part of the whole process.**

3. How do companies with sample databases determine that their assets include data assets? What percentage of data assets? Please elaborate.

**Response: Actually, companies keep these records for some regularity or obligatory conditions with no attention to their value of them. However, the currently held databases are a part of the data assets. How much portion of the data asset is not computed for the sample data?**

4. In assessing the valuation of data assets of listed companies in China from 2015-2023, the final valuation results were not seen using the data asset valuation model created based on the productive artificial intelligence model created in this thesis. Please add.

**Response: Significant variables are found that play a critical role in the data asset valuation model. Return on Assets (ROA), Return on Equity (ROE), and Operating Cash Flow (OCF) are found. Besides, in the conclusion section, these findings are stated in 2) verbally.**

Reviewer #2: The article highlights the dynamic nature of data assets and the need for more adaptive valuation models in the digital economy.

The proposed framework (integrating data feature extraction, value generation algorithms, and market adaptability assessment), offers a comprehensive solution for more precise and responsive valuation. Additionally, the discussion on how Generative AI enhances data valuation, through synthetic data generation, improved model training, and market adaptability, provides valuable insights into its broader implications. The introduction of a systematic framework for dynamic pricing, along with its potential applications for businesses and policymakers, makes this study a relevant contribution to the field of digital transformation and data asset management.

This article offers important perspectives on leveraging data as a strategic business asset. However, the empirical data collection process is not clearly explained, and the data itself is not yet accessible. It is only mentioned that "The sample primarily originated from Chinese 165 A-share listed companies, covering the period from 2015 to 2023" (lines 164-165) and "The calculation of data asset value was based on corporate financial statements and industry analysis reports" (Line 182).

A more detailed explanation of the data collection process is necessary to ensure transparency and enhance the credibility of the study. While the article references Chinese A-share listed companies as the dataset, it does not specify the exact sources of this data and other listed variables.

It would be beneficial to specify the exact source of the A-share listed companies used in the study. Providing this information, along with access to the data, would offer greater transparency and allow for a more thorough evaluation of the model’s robustness.

Additionally, making the data available would enhance the study’s replicability and reproducibility, further strengthening its impact. Clarifying the origin of the dataset would help readers assess the reliability and scope of the data used in model validation.

**Response: We provide the link for the data set:** [**https://www.hkex.com.hk/Mutual-Market/Stock-Connect/Eligible-Stocks/A-share-Lookup-Tools?sc_lang=en**](https://www.hkex.com.hk/Mutual-Market/Stock-Connect/Eligible-Stocks/A-share-Lookup-Tools?sc_lang=en)**. However, we do not share the collected and cleansed data set in the article, instead the data set was shared in China macroeconomic database.**

6. PLOS authors have the option to publish the peer-review history of their article ([what does this mean?](https://journals.plos.org/plosone/s/editorial-and-peer-review-process#loc-peer-review-history)). If published, this will include your full peer review and any attached files.
If you choose “no”, your identity will remain anonymous, but your review may still be made public.
**Do you want your identity to be public for this peer review?** For information about this choice, including consent withdrawal, please see our [Privacy Policy](https://www.plos.org/privacy-policy).

Reviewer #1: No

Reviewer #2: No

**Response: No responses are required.**
