# Supplementary material for: Data asset valuation model based on generative artificial intelligence
Source: PLoS One. 2025 Aug 18;20(8):e0328926. doi: 10.1371/journal.pone.0328926 (PMC12360564; doi:10.1371/journal.pone.0328926)
Supplement: Attachment — Submitted filename: MH250616-Response Letter 0615.docx [file pone.0328926.s002.docx]

**Response to Reviewers**

Reviewers' comments:

Reviewer's Responses to Questions

**Comments to the Author**

1. If the authors have adequately addressed your comments raised in a previous round of review and you feel that this manuscript is now acceptable for publication, you may indicate that here to bypass the “Comments to the Author” section, enter your conflict of interest statement in the “Confidential to Editor” section, and submit your "Accept" recommendation.

Reviewer #1: All comments have been addressed

Reviewer #2: All comments have been addressed

2. Is the manuscript technically sound, and do the data support the conclusions?

The manuscript must describe a technically sound piece of scientific research with data that supports the conclusions. Experiments must have been conducted rigorously, with appropriate controls, replication, and sample sizes. The conclusions must be drawn appropriately based on the data presented.

Reviewer #1: Yes

Reviewer #2: Yes

3. Has the statistical analysis been performed appropriately and rigorously?

Reviewer #1: Yes

Reviewer #2: Yes

4. Have the authors made all data underlying the findings in their manuscript fully available?

The [PLOS Data policy](http://www.plosone.org/static/policies.action#sharing) requires authors to make all data underlying the findings described in their manuscript fully available without restriction, with rare exceptions (please refer to the Data Availability Statement in the manuscript PDF file). The data should be provided as part of the manuscript or its supporting information, or deposited in a public repository. For example, in addition to summary statistics, the data points behind means, medians, and variance measures should be available. If there are restrictions on publicly sharing data—e.g. participant privacy or use of data from a third party—those must be specified.

Reviewer #1: Yes

Reviewer #2: No

**Response to Reviewer #2: We provide the link for the implemented dataset in the manuscript. Also, we provide a brief explanation for how the total dataset is reduced to the final form to be used in the modeling process. However, we do not share the preprocessed dataset. If requested by readers via email to the corresponding author, we can email it in unprocessed form, since preprocessing is an important part of the modeling process and a problem-specific aspect.**

5. Is the manuscript presented in an intelligible fashion and written in standard English?

PLOS ONE does not copyedit accepted manuscripts, so the language in submitted articles must be clear, correct, and unambiguous. Any typographical or grammatical errors should be corrected at revision, so please note any specific errors here.

Reviewer #1: Yes

Reviewer #2: Yes

6. Review Comments to the Author

Please use the space provided to explain your answers to the questions above. You may also include additional comments for the author, including concerns about dual publication, research ethics, or publication ethics. (Please upload your review as an attachment if it exceeds 20,000 characters)

Reviewer #1: (No Response)

Reviewer #2: My recommendation is minor: the manuscript should include detailed information about the data source and the specific steps taken in data collection and preparation, including any filters applied and the data cleansing process. While the cleaned dataset itself does not need to be shared, a clear explanation of the procedures followed is necessary.

In line 179, the authors mention that the data are acquired from Chinese A-share listed companies. However, the explanation is very minimal. Since the data is publicly available and the source has been stated in the response letter (https://www.hkex.com.hk/Mutual-Market/Stock-Connect/Eligible-Stocks/A-share-Lookup-Tools?sc_lang=en), I recommend that the authors also include this citation in the manuscript. This addition is important for clarity, transparency, and reproducibility of the study.

**Response: Lines 163-167 include how the dataset is filtered out. Since we apply strict rules to eliminate problematic data, for instance, missing observations and outliers are out completely, the final dataset obtained is composed of directly usable data in the modeling process.**  **The second sentence is also added to the text.**

7. PLOS authors have the option to publish the peer review history of their article ([what does this mean?](https://journals.plos.org/plosone/s/editorial-and-peer-review-process#loc-peer-review-history)). If published, this will include your full peer review and any attached files.


If you choose “no”, your identity will remain anonymous but your review may still be made public.


**Do you want your identity to be public for this peer review?** For information about this choice, including consent withdrawal, please see our [Privacy Policy](https://www.plos.org/privacy-policy).

Reviewer #1: No

Reviewer #2: Yes: Muhamad Prabu Wibowo
